# Biocompatible and Antimicrobial Cellulosic Support via Bioactive Emulsion-Based Film

**DOI:** 10.3390/polym18091067

**Published:** 2026-04-28

**Authors:** Angela Danila, Laura Chirila, Carmen-Mihaela Popescu, Ionela Cristina Voinea, Cristina-Mihaela Rimbu, Gizem Ceylan Türkoğlu, Emil-Ioan Muresan, Mariana Costea

**Affiliations:** 1 Faculty of Industrial Design and Business Management, “Gheorghe Asachi” Technical University of Iasi, 29 Prof. Dr. Docent D. Mangeron Blvd, 700050 Iasi, Romania; angela.danila@academic.tuiasi.ro; 2 Centre for Research and Innovation in Textiles and Fashion Industry—SMART-Tex-IS, Corp TEX 4, 29 Prof. Dr. Docent D. Mangeron Blvd, 700050 Iasi, Romania; 3 The National Research-Development Institute for Textiles and Leather Research, 6 Lucretiu Pătrășcanu Str., 030508 Bucuresti, Romania; laura.chirila@incdtp.ro; 4 Petru Poni Institute of Macromolecular Chemistry of the Romanian Academy, 41A Grigore Ghica Voda Alley, 700487 Iasi, Romania; 5 School of Computing, Engineering and the Built Environment, Edinburgh Napier University, Unit 1, Seven Hills Business Park, 37 Bankhead Crossway South, Edinburgh EH11 4EP, UK; 6 Faculty of Biology, University of Bucharest, 91–95 Splaiul Independentei, 050095 Bucharest, Romania; ionela-cristina.voinea@bio.unibuc.ro; 7 Faculty of Veterinary Medicine, Iasi University of Life Sciences, 8 Mihail Sadoveanu Alley, 707027 Iasi, Romania; cristina.rimbu@iuls.ro; 8 Department of Textile Engineering, Faculty of Engineering, Dokuz Eylül University, Central Campus, 35397 Izmir, Türkiye; gizem.turkoglu@deu.edu.tr; 9 ”Cristofor Simionescu” Faculty of Chemical Engineering and Environmental Protection, “Gheorghe Asachi” Technical University of Iasi, 73 Prof. Dr. Docent D. Mangeron Blvd, 700050 Iasi, Romania; emil-ioan.muresan@academic.tuiasi.ro

**Keywords:** O/W emulsion, Origanum, thyme, pectin, biocompatibility, antimicrobial test

## Abstract

Due to biodegradability, functionalization, and sustained release, polymer-based films are widely used in different industries. This study explores a bioactive emulsion-based film obtained using high-methoxy pectin (HMP), *Origanum onites* L. essential oil, and a hydroalcoholic extract of *Thymus vulgaris* L., prepared using various emulsion recipes. The emulsions obtained were applied to cellulose supports intended for topical applications. Bioactive textiles were analyzed using SEM-EDS elemental mapping, ATR FT-IR spectroscopy, biocompatibility assessment, antimicrobial activity assays, and analysis of comfort indices. SEM images of textile supports treated with bioactive emulsions confirmed the creation of a film surface and that the homogeneity of the film increases with increasing amount of glycerin, which acts as a plasticizer. Infrared spectra combined with their second derivatives and PCA indicate the presence of oregano essential oil, thyme extract, and pectin on the surface of the cotton. The biocompatibility evaluation of functionalized cotton supports revealed minimal cytotoxic effects on HaCaT human keratinocytes after 24 h of exposure. The results of the analyses showed that bioactive textile supports also exhibit antimicrobial activity. Therefore, the active emulsions with pectin, oregano essential oil, and hydroalcoholic extract of thyme provide biocompatible and antimicrobial active films by applying on cellulosic supports.

## 1. Introduction

Among natural resources, plant-based compounds have been recognized as reliable, sustainable options. In the textile field, these substances replace synthetic chemicals, providing fabrics with eco-friendly dyeing and additional skincare properties that simultaneously support both environmental concerns and human health [1,2,3,4,5,6,7,8,9]. Plants in the Lamiaceae family include approximately 236 genera and over 6000 species. Most species are aromatic and contain essential oils, which are primarily found in the leaves and are utilized in various fields, including cosmetics, food, perfumery, and pharmaceuticals. Numerous studies have reported the beneficial effects of Lamiaceae species on the human body, such as antioxidant, antimicrobial, and anti-inflammatory properties. Among these species are oregano (*Origanum onites* L.) and thyme (*Thymus vulgaris* L.). Each species contains a complex mixture of bioactive compounds, contributing to the overall bioactivity of the plant and thus being suitable for practical applications in food, health care, and biotechnology [10,11,12]. Due to their content of essential oils and polyphenolic compounds, these 2 species are suitable for practical applications in food, health care, and biotechnology. In recent years, increasing attention has been given to their antimicrobial, antioxidant, and anti-inflammatory activities, which open perspectives for multiple uses [13,14,15,16,17,18]. For this reason, oregano (*Origanum onites* L.) and thyme (*Thymus vulgaris* L.) species are used in several different ways, most often as bioactive extracts, essential oils, or even as emulsions. In this respect, these formulations expand the plant’s potential value beyond a single field.

Research studies on pectin-based bioactive emulsions focus mainly on the development of biodegradable films for obtaining packaging used in the food industry, due to pectin’s biodegradability, biocompatibility, and ability to improve dispersion and controlled release of active compounds [19,20,21].

However, most of these studies are still focused on food packaging and biofilms, and direct application to textile substrates is insufficiently explored. Available studies show that oregano and thyme are among the most active aromatic systems because their efficacy is linked mainly to phenolic terpenes such as carvacrol and thymol, and some non-textile studies report synergistic or additive effects when oregano and thyme essential oils are combined. In particular, oregano/thyme combinations have shown synergistic antibacterial activity in checkerboard and model-system studies, and more recently have been incorporated into pectin-containing bioactive films [22,23,24,25]. At the same time, no studies were identified that specifically address the combined use of oregano essential oil and thyme alcoholic extract as a single antimicrobial system for finishing textile supports. Thus, this study addresses an insufficiently explored aspect: the combination of two complementary bioactive sources (essential oil rich in volatile compounds and alcoholic extract rich in polyphenols) to obtain a biocompatible and antimicrobial cellulosic support, designed for single-use topical (skincare) applications.

## 2. Materials and Methods

### 2.1. Materials

The oregano essential oil used in this study was kindly donated by Doğal Destek Ürünleri Araştırma Sanayi ve Ticaret A.Ş. (Tabia Botanicals, Pure Nature), Aydın, Türkiye. The essential oil of oregano is extracted using water vapor distillation from *Origanum onites* L. plants grown in the South and Central Aegean regions, commonly known as pot marjoram. The main chemical compounds identified in oregano essential oil were 61.1% carvacrol, 10.5% linalool, 6.0% p-cymene, 4.4% γ-terpinene, 2.4% β-Bisabolene, and 2.1% thymol [26]. The hydroalcoholic extract of thyme leaves was obtained by ultrasonication (UP100H). Pectin from citrus peel (P9135, galacturonic acid ≥ 74.0%) was purchased from Sigma-Aldrich (Merck Life Science A/S), Søborg, Denmark. According to the Certificate of Analysis (lot number—SLBV5461), the pectin used in this study is a high-methoxyl pectin with 6% moisture, 85.3% (dried basis) galacturonic acid, and 7.7% (dried basis) methoxy groups. Polysorbate 80 (Tween 80), a cosmetic-grade non-ionic surfactant, was purchased from Sanflora, Romania. Vegetable glycerin (purity of 99.5%) was purchased from Herbavit, Oradea, Romania. All other chemical reagents used for analytical analysis were of analytical purity.

The cotton fabric used in this study was kindly donated by a local textile company. The textile fabric was 100% cotton with a plain-weave structure and a surface density of 130 g/m^2^.

#### 2.1.1. Preparation of O/W Emulsions

Citrus pectin was used as the continuous phase of the O/W emulsions. Polysorbate 80 was used as an emulsifying agent. Glycerol was used to provide thermoplasticity and to allow film casting [27]. The concentration ranges for pectin, glycerin, and active compounds were established based on preliminary tests. Pectin was adjusted to ensure film formation, glycerin to ensure plasticizing effect and homogeneity, and active compounds to obtain antimicrobial activity without affecting the stability of the emulsions.

In the first stage, citric pectin was dissolved in deionized water at 25 °C using a mechanical stirrer (stirring speed of 1000 rpm and 10 mm stirring paddle) for 1 h. In the second stage, the aqueous phase was obtained by mixing the aqueous solution of pectin, vegetable glycerin, and the hydroalcoholic extract of thyme leaves (the final volume of the emulsion was completed with a 50:50 hydroalcoholic extract of thyme leaves, which partially replaced the aqueous phase in the standard recipe). In stage 3, the oil phase was obtained by mixing thyme essential oil with Polysorbate 80 at 25 °C for 10 min using a magnetic stirrer. In stage four, the oil phase is dripped over the aqueous phase, and the mixture is stirred for 30 min at 25 °C using digital mechanical stirring. A 50:50 hydroalcoholic extract of thyme leaves was prepared by ultrasonication, at an amplitude of 40%, for 15 min, using a duty cycle of 0.5.

The recipes are presented in Table 1.

#### 2.1.2. Formation of the Emulsion Film on the Cellulose Support

Samples of cotton fabrics were dried at 100 °C for 3 min and weighed. In the first stage, the cellulosic support (previously washed and bleached) was immersed in 2 mL of 3% CaCl_2_ solution at room temperature and then dried. In the second stage, the emulsions obtained according to the recipes presented in Table 1 were applied with a squeegee onto cellulosic supports placed in Petri dishes and allowed to dry overnight at room temperature.

#### 2.1.3. Emulsions Characterization

##### Microscopic and Visual Analysis

Freshly prepared O/W emulsions were transferred into test tubes and then stored at room temperature (25 °C) for 12 days.

The emulsions obtained according to the working recipes were visualized using an optical microscope at 4× and 40× optical zoom (Bresser Biolux Touch Digital Microscope—Bresser GmbH, Rhede, Germany).

##### Storage Stability

The emulsions were stored as described in Section Microscopic and Visual Analysis. Changes in their appearance were monitored at 2-day intervals to indicate their storage stability. The creaming index was calculated using the relationship [28]:

(1) $$Creaming\;index\left(\%\right)=\frac{H}{H_{0}}\times 100$$

where:

H—the height of the separated water layer;
H_0_—the total emulsion height.

#### 2.1.4. Bioactive Textiles Analysis

##### SEM-EDS Elemental Mapping

Morphological and elemental characterization was performed by scanning electron microscopy (SEM) coupled with energy-dispersive X-ray spectroscopy (EDS). Analyses were carried out using an FEI Quanta 200 scanning electron microscope (FEI Company, Hillsboro, OR, USA), equipped with an EDAX Genesis EDS detector (AMETEK Inc., Mahwah, NJ, USA). Data acquisition and analysis were performed using EDAX Genesis software.

##### Coating Mass per Unit Area Analysis

Coating mass per unit area (g/m^2^) was calculated gravimetrically according to the ISO 2286-2:2016 standard [29], by calculating the difference between the mass per unit area of the treated samples and the mass per unit area of the untreated samples.

##### ATR FT-IR Spectroscopy

ATR infrared spectra have been recorded on an Alpha Bruker FT-IR spectrometer equipped with a diamond crystal, with a resolution of 4 cm^−1^, in the 4000–400 cm^−1^ region. For each sample, a number of 10 spectra were recorded, and further, the average spectrum was used for the analysis. Processing of the spectra was performed using the OPUS 7.5 program.

##### Biocompatibility Assessment

Human keratinocytes (HaCaT cell line) were cultivated in Dulbecco’s Modified Eagle Medium (DMEM, Sigma-Aldrich, St. Louis, MO, USA) supplemented with 10% fetal bovine serum (FBS, Gibco, Grand Island, NY, USA) at 37 °C in a 5% CO_2_-humidified atmosphere to assess the biocompatibility of textiles treated with oregano essential oils.

Initially, fabric extracts were obtained following the methodology outlined by Fanizza et al. [30]. Small textile fragments (0.3 cm × 0.3 cm) were sterilized by exposure to UV light for 24 h and then immersed in 1 mL of DMEM enriched with fetal bovine serum and agitated continuously at 240 rpm for 72 h. Meanwhile, human keratinocytes were seeded into 96-well plates at a density of 3 × 10^4^ cells per well and left to adhere overnight. Subsequently, each well was treated with 200 µL of the prepared fabric extracts for 24 h. The selected 24 h exposure period also reflects the realistic contact time expected for the intended biomedical applications, where prolonged skin contact is unlikely.

Cell viability was evaluated using the MTT assay [3-(4,5-dimethylthiazol-2-yl)-2,5-diphenyltetrazolium bromide], which relies on the enzymatic conversion of MTT into a purple-colored formazan compound. Nitric oxide (NO) levels were quantified via the Griess reagent-based colourimetric technique, measuring absorbance at 550 nm after mixing the culture medium with the reagent in a 1:1 ratio. To assess membrane integrity following fabric exposure, lactate dehydrogenase (LDH) release was analyzed. For this, 50 µL of the cell culture supernatant was combined with an equal volume of dye and catalyst solution from the Cytotoxicity Detection Kit (Roche, Mannheim, Germany) and incubated for 30 min at room temperature. Absorbance was then recorded at 490 nm using the FlexStation 3 microplate reader (Molecular Devices, San Jose, CA, USA). Statistical analysis was performed through one-way ANOVA followed by Bonferroni’s post hoc test, with *p*-values below 0.05 considered statistically significant.

The LIVE/DEAD™ assay kit (Invitrogen, Thermo Fisher Scientific, Waltham, MA, USA) was used following the manufacturer’s protocol to observe the spatial distribution of viable and non-viable cells after 24 h of exposure to fabric extracts. Fluorescent images were acquired using an Olympus IX71 inverted microscope (Olympus, Tokyo, Japan).

##### Antibacterial Activity Assay

Antibacterial testing of the samples was carried out on two bacterial species: Staphylococcus aureus ATCC and Escherichia coli ATCC, using the agar diffusion technique. The principle of the test consists of evaluating the antibacterial effect induced by the tested sample through measurement of the inhibition zones formed around the test specimens (distance between the edge of the sample and the edge of visible microbial growth).

Bacterial suspensions were prepared by resuspending bacterial colonies from 24 h cultures in physiological saline and adjusted to a cell density of 1.5 × 10^8^ CFU/mL. Using a sterile swab, a 100 µL aliquot of the microbial suspension was spread by streaking over the surface of Mueller–Hinton nutrient agar (Oxoid), previously distributed in sterile Petri dishes (90 mm). After drying for 5 min at room temperature, the textile samples, cut to approximately 1 cm^2^, were placed on the agar surface.

The samples were incubated for 24 h at 37 °C, and the results were interpreted based on the dimensions (mm) of the microbial inhibition zones measured with a millimeter ruler. For increased accuracy, the test was performed in triplicate, and the arithmetic mean and standard deviation were calculated.

##### Antifungal Activity Assay

The eight textile materials (seven functionalized and one control) were tested according to the ASTM G21-15 standard [31] method. This is a standard test method developed by ASTM International (formerly the American Society for Testing and Materials) for evaluating the resistance of materials to fungal growth. The purpose of this test is to determine the potential of a material to support fungal growth under controlled laboratory conditions.

The test involves exposing the material under investigation to a fungal spore suspension and providing optimal conditions for fungal growth, including temperature, humidity, and nutrient availability. During the test, the material is either placed in direct contact with the fungal suspension or positioned in proximity to the spore source. The test specimens are incubated for a specific duration, depending on the fungal species used for the assay. After the incubation period, the samples are visually examined for any signs of fungal growth, such as discoloration, biofilm formation, or hyphal development. The ASTM G21-15 method provides guidelines for evaluating fungal growth on test specimens and assigning a qualitative rating based on the extent of growth observed. The ratings range from 0 (no fungal growth) to 4 (heavy fungal growth), allowing for a comparative assessment of the antifungal performance of materials. It is important to note that ASTM G21-15 does not provide quantitative measurements of fungal resistance, nor does it indicate the specific fungal species that may be encountered under real-world conditions. Instead, it serves as a reference protocol for evaluating the general susceptibility of materials to fungal growth and for comparing their relative performance.

The materials were tested against the pathogenic strain Candida albicans on Sabouraud agar medium, in Petri dishes with a diameter of 90 mm. After pouring the medium into the plates, 0.8 mL of the undiluted fungal inoculum stock solution was spread over the surface using a Drigalski spatula. The materials were sampled into disks with a diameter of 4.5 cm and placed in the center of the plates. The samples were incubated for 96 h at 37 °C.

##### Variations in Color of Bioactive Textiles

The application of emulsions containing hydroalcoholic thyme extract onto bleached cotton textile supports led to a change in the color of the obtained samples, not only in the shade of the same color. Thus, the color modification was evidenced by means of the color parameters (L, a, b*), the color strength determined using the Kubelka–Munk equation, and the color difference (∆E) [32].

(2) $$\frac{K}{S}=\frac{{\left(1-R\right)}^{2}}{2R}$$

where:

R—reflectance;
K—absorption coefficient;
S—scattering coefficient.

The color difference was determined using the following equation [33]:

(3) $$∆E=\;\sqrt{{\left(∆L^{*}\right)}^{2}+{\left(∆a^{*}\right)}^{2}+{\left(∆b^{*}\right)}^{2}}$$

where:

L*—luminance (it ranges from 0 to 100);
a*—green–red axis;
b*—blue–yellow axis.

The values obtained are the average of five readings taken at different points on the surface of the textile support.

##### Analysis of Comfort Indices

Comfort indices were tested by analyzing air and vapor permeability and hygroscopicity. The air permeability was determined according to the SR EN ISI 9237 Standard [34] using a METEFEM (Budapest, Hungary) apparatus with a surface test area of 5.0 cm^2^ and with a pressure drop of 100 Pa. Hygroscopicity was determined according to the EN ISO 12571:2000 Standard [35], and water vapor permeability according to the ISO 11092:1993 Standard [36].

## 3. Results

### 3.1. Emulsion Characterization

#### 3.1.1. Visual and Microscopic Evaluation of Emulsions

**Figure 1:**
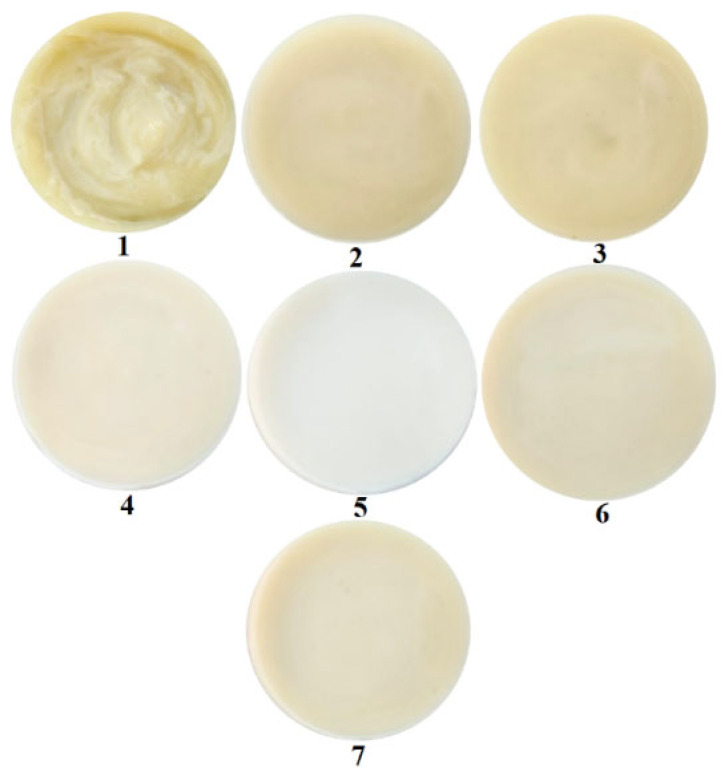
Visual appearance of emulsions.

**Figure 2:**
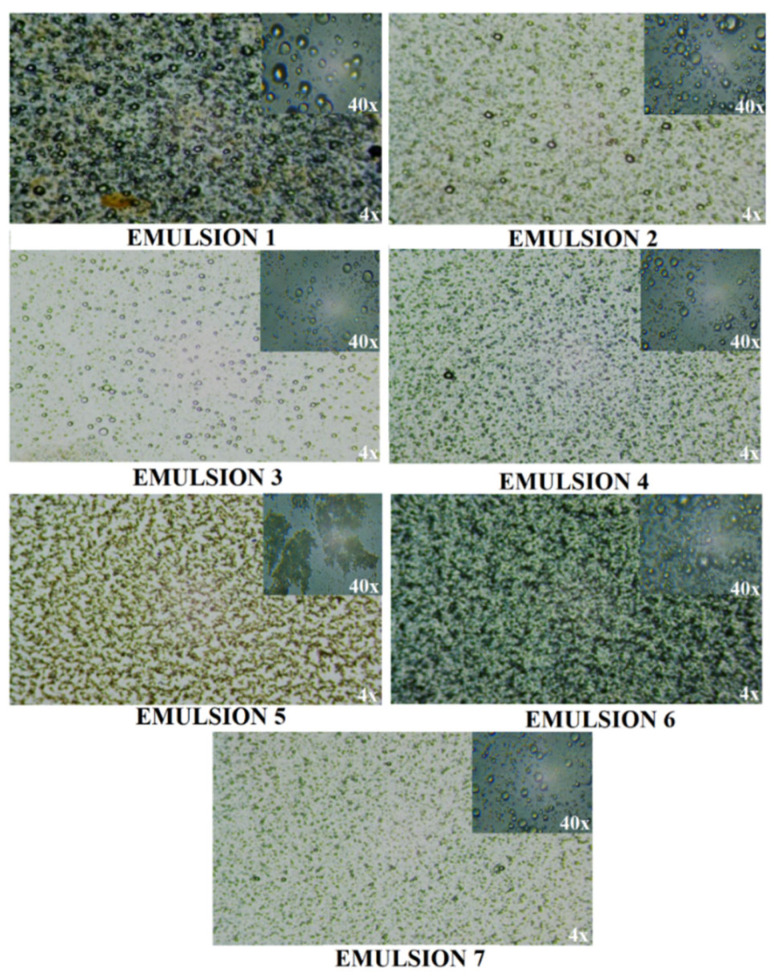
Microscopic images of the emulsions, immediately after preparation.

Visual and microscopic images of the seven emulsions obtained are shown in Figure 1 and Figure 2.

According to the images shown in Figure 1, emulsions 1–7 present a uniform appearance, without variations, and are opaque. The visual texture is fine, without visible aggregations. Emulsion 1 presents a viscous semi-solid consistency, and emulsions 2–7 present a less viscous semi-solid consistency. The color of the emulsions is given by the presence of the hydroalcoholic extract of thyme leaves and varies in a range between white (the emulsion 5 that does not contain a hydroalcoholic solution of thyme leaves) and cream-yellow.

From the analysis of the microscopic images, it results that the emulsion that does not contain the hydroalcoholic extract of thyme presents a diffuse aggregation, without a clear delimitation of the globules. With the addition of the hydroalcoholic extract of thyme, it is observed that it presents well-defined globules.

**Figure 3:**
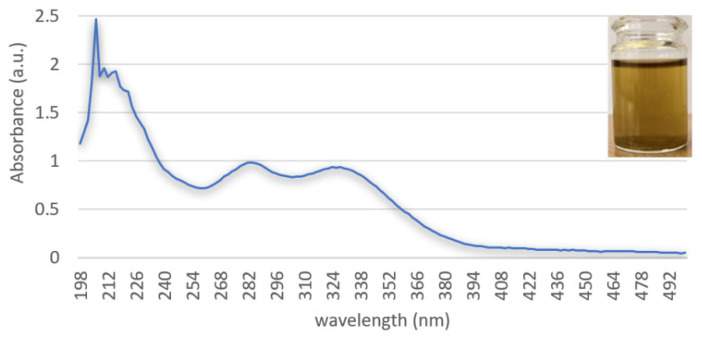
UV-Vis spectra of the hydroalcoholic extract of thyme leaves.

The explanation can be given by the presence of phenols (Figure 3) in the hydroalcoholic extract of thyme leaves, which presents emulsification ability and thus has the role of cosurfactant in the creation of stable emulsions and helps in the uniform distribution of the internal phase [37].

UV-Vis spectral analysis indicates that the hydroalcoholic extract from thyme leaves is characterized by the presence of a very intense absorption band in the wavelength range of 200–220 nm, which is specific to compounds containing heteroatoms with nonbonding electron pairs and non-conjugated double bonds. In addition, the extract from thyme leaves is also characterized by the presence of a fairly intense absorption band around 284 nm, indicating the presence of flavonoids in its composition [38]. The absorption band at 284 nm indicates the presence of an aromatic ring substituted with a hydroxyl group, likely due to a *π-π* electronic transition [15]. The absorption band at 324 nm corresponds to the presence of rosmarinic acid, the major phenolic compound in thyme leaves, as also confirmed by the literature (λmax for rosmarinic acid hexoside = 322 nm; λ_max_ for cis-rosmarinic acid = 328 nm) [39].

#### 3.1.2. Storage Stability

The seven prepared emulsions were stored at room temperature and analyzed over a period of 14 days. The calculated creaming index is presented in Table 2.

According to the results presented in Table 2, the creaming index of the emulsions analyzed over a period of 14 days was 100%, indicating that the emulsions are stable. Citric pectin also played an important role in their stability, acting as a thickening agent and stabilizer.

### 3.2. Functional Textile Supports Characterization

Intermolecular interactions play a key role in the stability of polymer networks, being frequently described as the result of the synergistic action of hydrogen bonds, metal coordination interactions, and ion–dipole interactions [40]. Similarly, in the investigated system, film formation can be attributed to an ensemble of interactions between the functional groups of the components. The different functional groups are involved in HMP film formation, such as deprotonated carboxyl groups (-COO-) and methylated carboxyl groups. In film formation, Ca^2+^ bridges, hydrogen bonding between the hydroxyl groups (-OH) of the polysaccharide chain of pectin and the hydroxyl groups of the glucose unit of cellulose, and hydrophobic interactions between methylated carboxyl groups of pectin can be involved [41].

Ca^2+^ ions act as a bridge between cellulose and pectin through ionic bonds and Lewis interactions (Ca^2+^ ions are considered good Lewis acids) [42].

**Figure 4:**
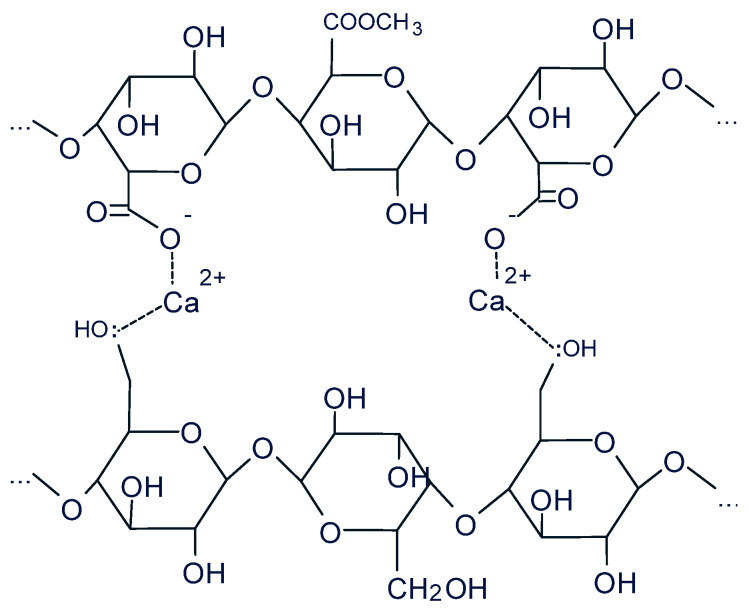
A possible intermolecular pectin/Ca^2+^/cellulose interaction.

The ionic bonds formed between calcium ions (Ca^2+^) and negative carboxyl groups (-COO^−^) are sufficiently stable to influence the structure of the polysaccharide network. In the case of Lewis interactions, the coordination bond is formed between Ca^2+^ ions and the oxygen (: O) of the –OH groups of cellulose (Figure 4).

#### 3.2.1. SEM-EDS Elemental Mapping

**Figure 5:**
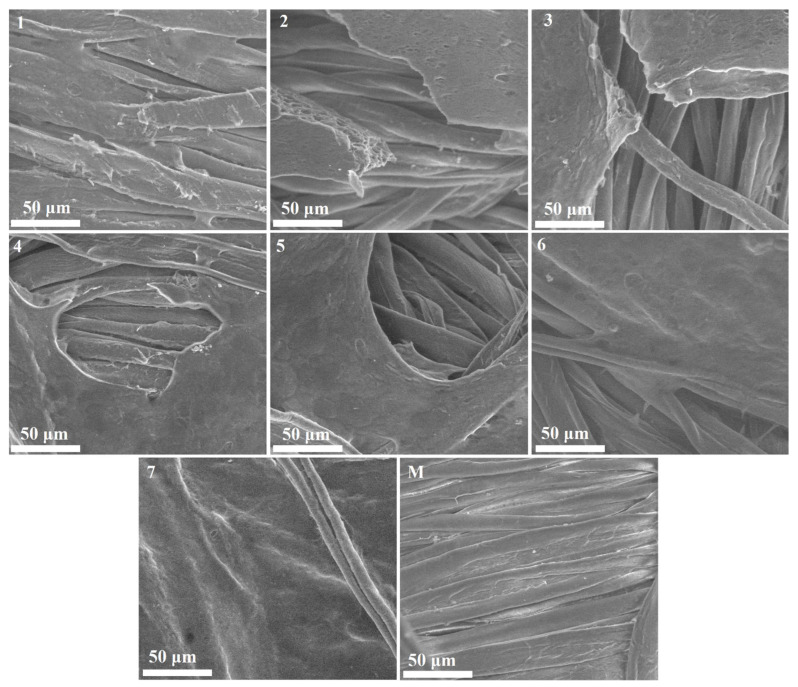
The SEM micrographs of the functionalized cotton samples at 2000× magnification.

The surface morphology of the samples was tested using SEM analysis, and their elemental composition was analyzed using EDS mapping. In the emulsions obtained and applied on cellulose supports, high methoxyl pectin acts as a matrix for embedding the essential oil and the other components, thus facilitating the apparent interaction with calcium ions, which appears to be a direct binding, in fact being mediated by the structure of the emulsion. According to the SEM images obtained and presented in Figure 5, the interaction between the 7 emulsions and calcium ions leads to the formation of a less homogeneous film, a result also confirmed by the specialized literature [43]. A possible cause of the inhomogeneous film may be the presence of methoxylated groups, which prevent uniform binding with calcium ions, resulting in a network with “gaps” or weaker areas. According to some studies in the specialized literature, in the case of using HMP, film formation is also due to hydrophobic interactions and hydrogen bonds, which are prone to local variations if the polymer formulation also contains other compounds [44] (essential oil, hydroalcoholic plant extract, and plasticizer).

According to the SEM images, the most homogeneous distribution of the pectin film is shown by samples 6 and 7, which shows that the homogeneity of the film increases with increasing amount of glycerin. Glycerin acts as a plasticizer (glycerin molecules intercalate between pectin chains), reducing the internal tension of the pectin network and allowing the film to stretch more evenly on the cellulose support, thus improving continuity and reducing voids in the resulting film [45].

**Figure 6:**
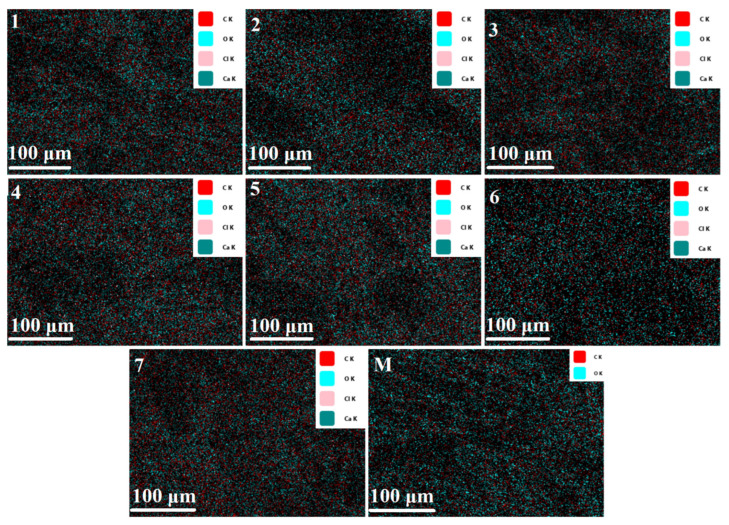
Mapping images of C, O, Cl, and Ca elements on the functionalized cotton samples.

Analysis of the functionalized cotton samples (samples 1–7) containing C, O, Cl, and Ca elements confirmed that all elements are uniformly distributed over the entire textile surface, indicating the homogeneity of the bioactive emulsions coating and its application. EDS mapping (Figure 6) revealed the presence of carbon and oxygen in both the control (M) and the functionalized cotton samples (1–7), with increased carbon content in the functionalized cotton samples, suggesting the presence of emulsions.

SEM images were processed using Fiji software (Fiji Is Just ImageJ), based on ImageJ version 1.54p, by applying a classification-based segmentation method, which allows differentiation between film-covered areas and uncovered fibers, taking into account both intensity and textural characteristics (Table 3).

#### 3.2.2. Coating Mass per Unit Area Analysis

The results obtained are presented in Table 4.

According to the results obtained, the coating mass per unit area varies between 14.601 ± 0.8 g/m^2^ for sample 2 and 32.285 ± 1.4 g/m^2^ for sample 7. These values reflect the total amount of emulsion applied per unit area and cannot be directly correlated with the layer thickness, since the surface morphological analysis (SEM analysis) revealed an inhomogeneous distribution of the polymeric film on the surface of the textile supports.

#### 3.2.3. ATR FT-IR Spectroscopy

**Figure 7:**
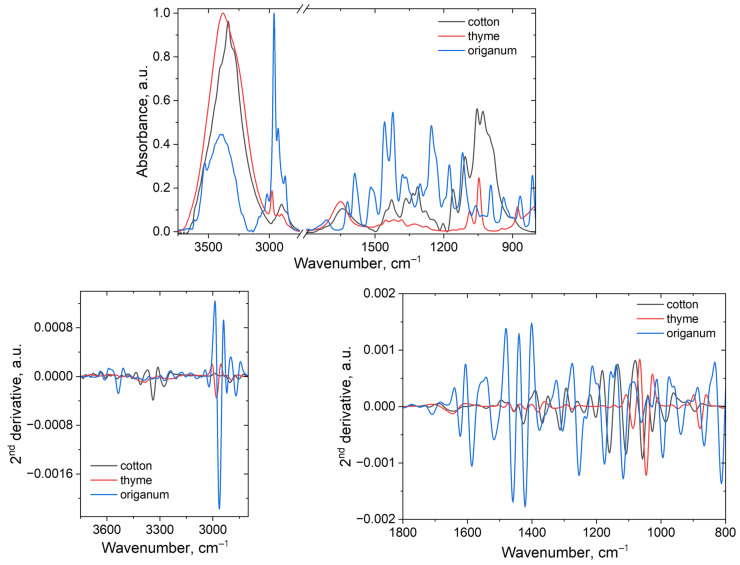
ATR FT-IR spectra of cotton fabric, hydroalcoholic extract of thyme leaves, and oregano (Origanum) essential oil.

The ATR FT-IR spectra of cotton, oregano essential oil, and hydroalcoholic extract of thyme leaves are represented in Figure 7.

The spectrum of cotton (black contour) is a typical spectrum of cellulose which presents two main regions, namely 3750–2800 cm^−1^ assigned to different OH stretching vibrations and hydrogen bonds as well as to CH_2_ and CH_3_ symmetric and asymmetric stretching vibrations and the 1800–800 cm^−1^ region (also called fingerprint region) assigned to different stretching and deformation vibrations of different groups belonging to cellulose. Thus, the OH stretching vibration large band is composed from several sub-bands, as seen from the second derivative spectrum: at 3542 cm^−1^ assigned to stretching vibration of weakly bound adsorbed water, at 3457 and 3407 cm^−1^ assigned to O2‒H2⋯O6 intramolecular hydrogen bonds stretching vibration, at 3340 cm^−1^ assigned to O5‒H5⋯O3 intramolecular hydrogen bonds stretching vibration, at 3276 cm^−1^ assigned to O6‒H6⋯O3 intermolecular hydrogen bonds stretching vibration for the Iβ and Iα cellulose forms. The bands assigned to antisymmetric and symmetric stretching vibration of the methyl and methylene groups were identified at 2957, 2903, and 2854 cm^−1^, respectively [45,46,47,48,49,50].

The second region (between 1800 and 750 cm^−1^) present main bands at around 1640 cm^−1^ assigned to OH deformation vibration of adsorbed water and conjugated C–O, 1456 and 1427 cm^−1^ assigned to C–H deformation vibration and O–H in plane bending, 1367, 1335 and 1312 cm^−1^ assigned to CH_2_ rocking vibration, 1276 cm^−1^ assigned to C–H bending mode, 1238 and 1160 cm^−1^ assigned to C–O–C stretching vibration mode of the pyranose ring, at 1202, 1108 and 986 cm^−1^ assigned to C–O stretching vibration, 1160 cm^−1^ assigned to C–O–C stretching vibration mode of the pyranose ring, 1057 cm^−1^ assigned to C–O stretching mainly from C(3)–O(3)H, 1024 cm^−1^ assigned to C–O and C–C stretching ring, 929 cm^−1^ assigned to pyranose ring stretching, and 896 cm^−1^ assigned to CH deformation vibration [46,47,48,49,50,51].

Main components in oregano essential oil are carvacrol, linalool, p-cymene, γ-tirpenene, β-bisabolene, and thymol, with a high concentration of carvacrol. The infrared spectrum (blue contour) presents in the first region a series of bands at 3535, 3388, 3271, and 3207 cm^−1^ assigned to hydroxyl group stretching vibration of phenolic and aliphatic OH groups and 3022, 2963, 2921, 2869 cm^−1^ assigned to symmetric and antisymmetric CH_2_ and CH_3_ stretching vibrations in monoterpenes [52,53,54,55]. In the fingerprint region, the oregano essential oil spectrum presents bands at 1660, 1621, 1587, 1516 cm^−1^ assigned to C=C stretching vibration of the aromatic ring and C=C in aliphatic structures, at 1458 and 1421 cm^−1^ assigned to CH_2_ bending (deformation) vibration, at 1377 cm^−1^ assigned to the isopropyl methyl group bending vibration, and at 1254 cm^−1^ assigned to C–O stretching vibration (phenolic). The region below 1200 cm^−1^ is assigned to C–O–C stretching vibration (in ether/alcohol), C–O stretching vibration, and C–H out-of-plane bending vibration. The bands from 1175, 1117, 994, 866, and 812 cm^−1^ are specific to the carvacrol compound [53].

The hydroalcoholic extract from thyme leaves is rich in phenolic compounds, phenolic acids, and flavonoids, with the major component being rosmarinic acid [56]. The infrared spectrum (represented by red contour) in the 3800–2800 cm^−1^ region indicate the presence of 3520, 3383, and 3249 cm^−1^ bands (in the second derivative spectrum) which are assigned to OH stretching vibration in rosmarinic acid, flavonoids, and other polyphenols and at 2979, 2933, and 2894 cm^−1^ assigned to CH in methyl and methylene groups from flavonoids, and sugars. In the second region, from the second derivative spectrum one can observe the following bands: at 1645 and 1549 cm^−1^ assigned to C=O (carboxylic/ester) and conjugated C=O, at 1524 cm^−1^ assigned to aromatic skeletal vibrations (C=C) (in flavonoids and phenolic acids), at 1487, 1454, and 1419 cm^−1^ assigned to CH_2_/CH_3_ bending/aromatic C–C, at 1382 cm^−1^ assigned to CH_3_ symmetric deformation, at 1274 cm^−1^ assigned to phenolic C–O stretch, at 1162, 1130, 1087, and 1045 cm^−1^ assigned to C–O–C and C–O stretching vibrations, at 947 cm^−1^ assigned to =C–H deformation/C–O–H bending vibration, and 808 cm^−1^ assigned to aromatic C–H out-of-plane bending vibration (specific to carvacrol and thymol) [57,58].

**Figure 8:**
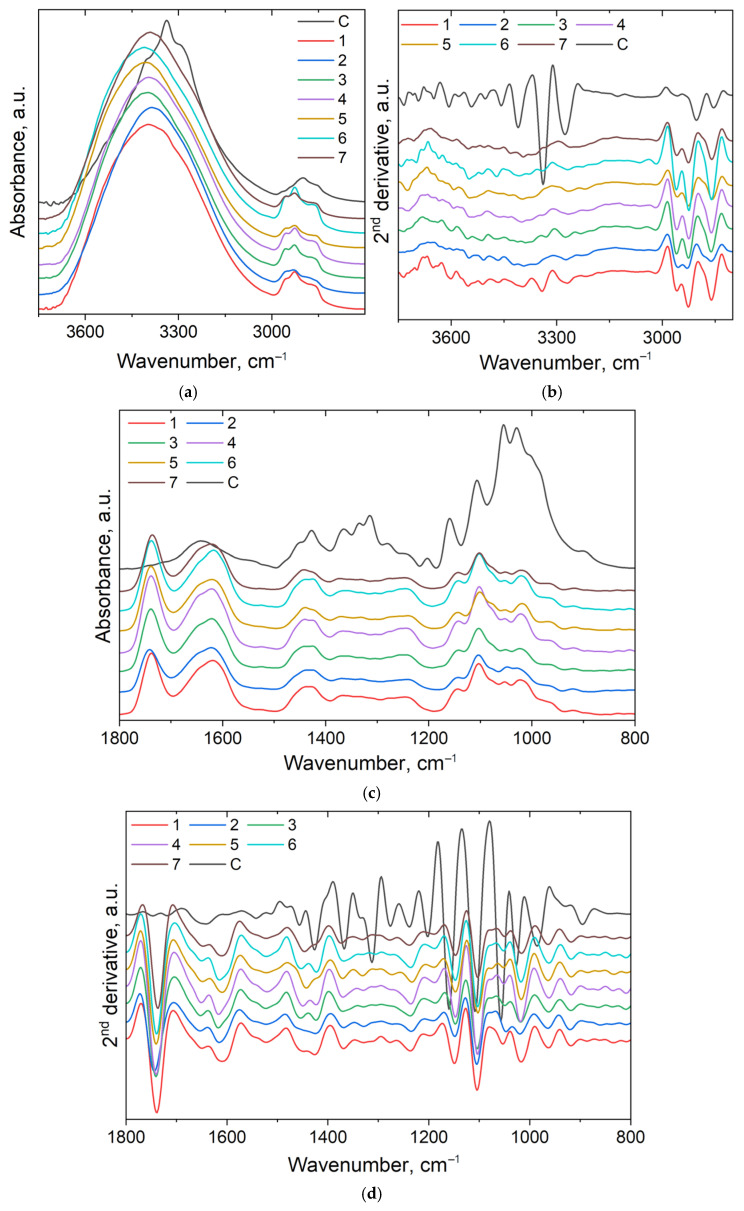
ATR FT-IR spectra (**a**,**c**) and their second derivatives (**b**,**d**) of the studied samples in the 3750–2800 cm^−1^ (**a**,**b**) and 1800–800 cm^−1^ (**c**,**d**) regions.

The ATR-FTIR spectra of the cotton material and modified cotton material, as well as their second derivatives, are presented in Figure 8.

In the first region (Figure 8a,b), the broad band from 3338 cm^−1^ in the control sample is shifted to 3399–3415 cm^−1^ in treated samples, and its shape resembles the shape of the band from thyme and pectin spectra. The shifting of the maximum of this band is due to the hydrogen bonds interactions taking place mainly between the pectin shell and cellulose. In the second derivative spectra, the band from 3457 cm^−1^ (cotton) is shifted to 3465–3470 cm^−1^ in the modified material, 3408 cm^−1^ (in cotton), 3387 cm^−1^ (in O) and 3384 (in T) appears at 3398–3395 cm^−1^ in the modified material indicating the modification of the hydrogen bonding architectures with formation of new intermolecular bonds between the OH groups from the components. Further in the spectrum of the control sample, there is a band with a maximum at 2899 cm^−1^ with shoulders towards both sides. From the second derivative spectrum, we could identify three distinct bands at 2958, 2902, and 2854 cm^−1^ assigned to symmetric and antisymmetric stretching vibrations of methyl and methylene groups. In treated cotton material, these bands are shifted to 2958–2961, 2924–2926, 2860 cm^−1^, indicating once again the presence of the treatment materials (especially: oregano essential oil, thyme extract, and pectin) on the surface of the cotton.

In the fingerprint region (Figure 8c,d), the spectra of the treated material present clear differences compared to the spectrum of the control sample, visible both from the spectra and their second derivatives. The treated samples present two medium-intensity bands at about 1738 and 1617 cm^−1^ assigned to the stretching vibration of ester (C=O) in the methyl- esterified carboxyl group (COO–R) and the C=O stretching vibration regarding the ionic carboxyl groups (COO−). The second band also overlaps with the bands present in oregano essential oil and thyme extract. Other differences can be observed as follows: the band from 1148 cm^−1^ is assigned to glycosidic (O–C–O) bond vibrations present in pectin (1144 cm^−1^ in [59]) as well as in oregano essential oil (1147 cm^−1^); the bands from 1053 and 1016 cm^−1^ as a combination from specific conformations around the glycosidic bonds of pectin, C–O–C and C–O stretching from oregano and thyme [59,60,61], but also formation of new C–O–C bonds.

**Figure 9:**
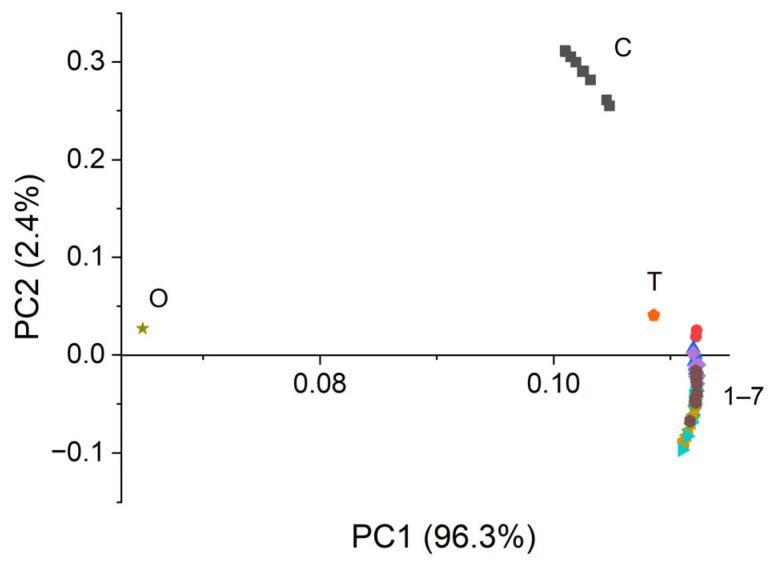
Principal component scores performed on infrared spectra in the 1800–750 cm^−1^ region for control and impregnated cotton fabrics, as well as oregano essential oil and hydroalcoholic extract of thyme leaves.

Principal component analysis (PCA) gives more detailed information on the differences between the series of the samples. In order to perform the PCA, the infrared spectra were used in 10 replicates for each sample. The PC scores—PC1 score versus the PC2 score—are plotted in Figure 9. The principal component factor 1 (PC1) describes 96.3% and principal component factor 2 (PC2) describes 2.4% of data variance, so 98.7% of the existing variances in the spectra can be captured using these two dimensions. The differences taking place between the control cotton sample and the impregnated samples can be clearly observed. Moreover, to see the correlation between the active compounds used in the emulsions and the impregnated cotton fabric, we also used the oregano essential oil and thyme leaves hydroalcoholic extract spectra. Even though PC1 describes 96.3% of the data variance and PC2 only 2.4%, it can be observed that PC2 is the most informative latent variable to differentiate between the samples. Therefore, both positive PC1 and PC2 values are observed for the control sample, while the impregnated samples present positive values for PC1 and negative and close to 0 values for PC2. There is no clear difference between the impregnated samples, only small variations. This is because there are small differences between the emulsion’s concentrations, all of them containing the same compounds. Further, it can be observed that thyme is very close to the treated samples, while oregano is close to 0 values for both PC scores.

This indicates that the thyme spectrum has a strong influence on the spectra of impregnated cotton samples.

Overall, infrared spectra combined with their second derivatives and PCA results indicate the presence of modifications in the hydrogen-bonding system of cellulose. The shifting of the bands from the OH stretching region indicates the formation of new intermolecular hydrogen bonds, mainly between the hydroxyl groups from cellulose and the hydroxyl/carboxyl groups from pectin and plant derived compounds. At the same time, the modifications of the bands from carbonyl/carboxyl region (1738 and 1617 cm^−1^) suggest the contribution of esterified and ionic carboxyl groups from pectin, involved in additional polar interactions with the cotton support. The changes in the 1148–1016 cm^−1^ region further indicate the contribution of glycosidic and C-O/C-O-C groups from pectin, oregano essential oil and thyme extract, confirming the present of the treatment material on the cotton surface and the establishment of intermolecular interactions between the components.

These observations are further supported by PCA, which reveals a clear separation between the control and treated samples. Although PC1 explains most of the spectral variance, PC2 proved to be more informative for the differentiation of the samples, separating the untreated cotton from impregnated samples. The position of thyme closer to the treated samples indicates that this extract has stronger spectral contribution to the spectral profile of the modified cotton, while the limited separations observed among the treated samples are consistent with their similar composition, differing only in the concentrations of the same active compounds.

#### 3.2.4. Evaluation of Fabric Extracts’ Biocompatibility on Human Keratinocytes

Given the increasing interest in using functionalized cotton supports via bioactive O/W emulsions for therapeutic and wound care applications, it is imperative to assess their biocompatibility on human skin cells. Since keratinocytes represent the primary cellular component of the epidermis, evaluating their response to these biofunctional textiles is essential to ensure safety, minimize cytotoxic effects, and validate their suitability for medical use.

**Figure 10:**
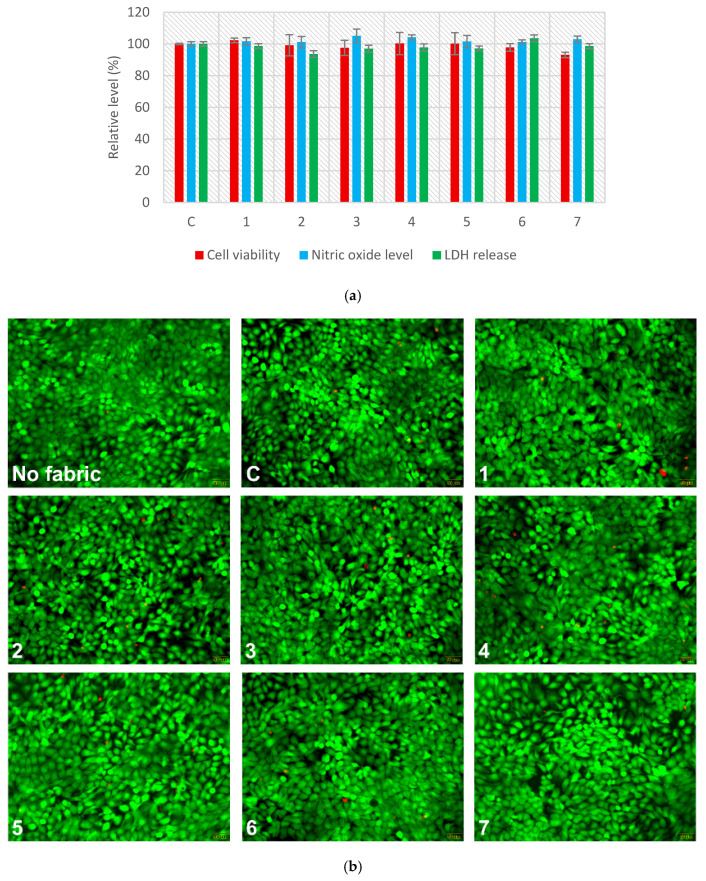
Biocompatibility evaluation of functionalized cotton supports on human keratinocytes (HaCaT cells) after 24 h of incubation: (**a**) Quantitative analysis of cell viability, nitric oxide production, and lactate dehydrogenase (LDH) release; (**b**) Fluorescence staining of live cells (green, calcein-AM) and dead cells (red, propidium iodide). All fluorescence images were captured using a 20× objective. Data are presented as mean ± standard deviation (*n* = 3), normalized to cells treated with extracts from untreated fabric (C). No statistically significant differences were observed between treated samples and control.

The biocompatibility evaluation of functionalized cotton supports revealed minimal cytotoxic effects on HaCaT human keratinocytes after 24 h of exposure (Figure 10a). Cell viability remained consistently high across all tested fabric samples, with values close to the control, indicating that the functionalized cotton supports did not compromise cellular integrity. Similarly, nitric oxide levels showed no significant elevation, suggesting an absence of inflammatory response. LDH release, a marker of membrane damage, also remained within control levels, further supporting the non-toxic nature of the tested textile supports.

Fluorescence imaging of live/dead cell staining (Figure 10b) further confirmed the absence of cytotoxic effects following 24 h of incubation with extracts from both untreated and emulsion-treated fabrics. Also, it is noteworthy that the untreated cotton woven fabric (sample C) did not impact cell viability or compromise membrane integrity under any exposure condition. Across all tested samples, the number of dead cells (red fluorescence) remained minimal and comparable to wells cultured without textile exposure. Collectively, these findings demonstrated that the emulsion-treated cotton fabrics exhibit favorable biocompatibility profiles, making them promising candidates for medical and dermatological applications.

Similar observations have been documented in prior studies, supporting the safe interaction between Origanum vulgare essential oil and human cells. For instance, Leyva-López et al. [62] showed that terpenes, like thymol and carvacrol, extracted from three Mexican oregano species (L. graveolens, L. palmeri, and H. patens) significantly decreased the production of reactive oxygen species (ROS) and NO in RAW 264.7 macrophage cells following stimulation with lipopolysaccharide (LPS). Another previous study using wool fabrics infused with oregano oil has demonstrated a significant increase in HaCaT cell viability (after 48 h exposure to concentrations of 25% and 12.5% fabrics extract in culture media), indicating a lack of cytotoxicity under those conditions [63]. Moreover, in vivo experiments conducted by the same group reported accelerated wound healing, enhanced collagen deposition, and improved antioxidant defenses in treated animals. Although those findings were based on wool substrates, our results using cotton fabrics similarly show high keratinocyte viability and minimal cytotoxicity, suggesting that the beneficial properties of emulsions enriched with thyme-derived products may be preserved across different textile carriers. This supports the broader potential of emulsions enriched with thyme-derived products-treated natural fabrics for biomedical applications, but further long-term and mechanistic evaluations will be important in subsequent stages of development.

#### 3.2.5. Antibacterial Activity Assay

**Figure 11:**
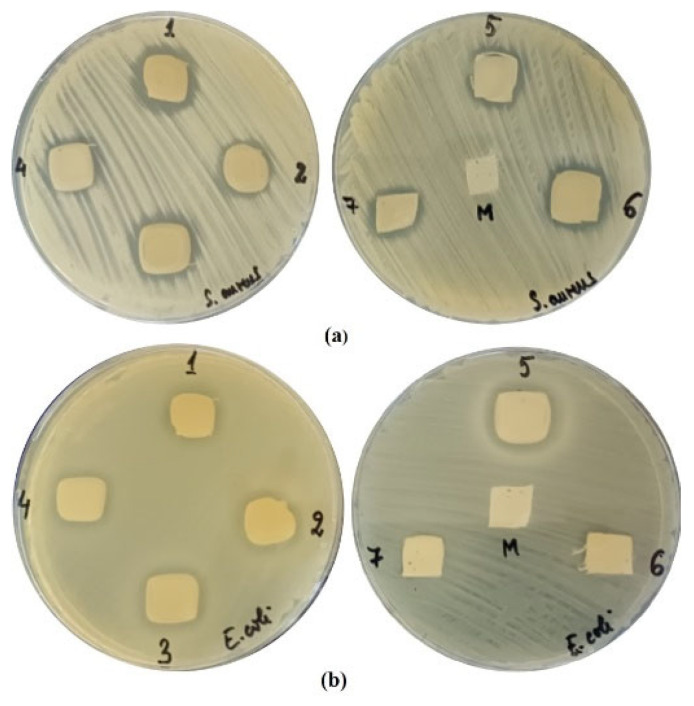
Antibacterial activity of the tested samples against: (**a**) *Staphylococcus aureus*; (**b**) *Escherichia coli.*

The results of antibacterial activity are presented in Figure 11 and Table 4.

The diameters of the inhibition zones obtained when testing the antimicrobial activity of textile samples against the bacterial species *Staphylococcus aureus* ATCC 25923 and *Escherichia coli* ATCC 25922 are presented in Table 5.

According to the results obtained and presented in Table 5, the antimicrobial activity is mainly due to the essential oil of oregano, which contains major compounds such as carvacrol, linalool, p-cymene, γ-terpinene, β-bisabolene, and thymol. The antibacterial effect of the hydroalcoholic extract of thyme leaves is evident only in the case of the sample treated with emulsion 1 (it contains the highest amount of hydroalcoholic extract). A possible explanation may be that the minimum concentration of polyphenolic compounds exhibiting antibacterial activity was reached. The experimental data obtained from the antibacterial testing of the other emulsions (emulsions 2–7) do not follow a clear trend and thus cannot be directly correlated with the analyzed variables, suggesting a possible complex or even antagonistic interaction between the active compounds in the hydroalcoholic extract and the oregano essential oil.

The main mechanism of antimicrobial action of essential oils is the increase in permeability and subsequent disruption of the plasma membrane. For example, carvacrol (the major compound in the composition of oregano essential oil—61.077%) can cross the bacterial plasma membrane and bind monovalent molecules or cations, transporting them outside the bacterial cell and thus being able to disrupt the function of plasma membranes [64,65].

Specialized literature confirms the antibacterial effect of oregano essential oil, which exhibits strong inhibitory activity against *S. aureus* and *E. coli* bacteria on textile substrates, and their effectiveness depends on the polymer matrix, the degree of loading, and the immobilization method [66,67]. Research confirms that the antimicrobial activity of oregano essential oil is primarily attributed to phenolic compounds (carvacrol and thymol) [68]. Carvacrol has been reported to exhibit stronger antibacterial activity than thymol, particularly against *S. aureus* and *E. coli* [69].

Some studies confirm that hydroalcoholic extracts from thyme leaves (*Thymus vulgaris*) exhibit antibacterial activity against Gram-positive bacteria, *Staphylococcus aureus*, and Gram-negative *Escherichia coli*. Phenolic compounds, such as rosmarinic acid, carvacrol, and thymol, contribute to the antibacterial effect through multiple mechanisms, including disruption of the bacterial membrane, inhibition of proton pumps, and reduction in biofilm formation [56,70].

#### 3.2.6. Antifungal Activity Assay

**Figure 12:**
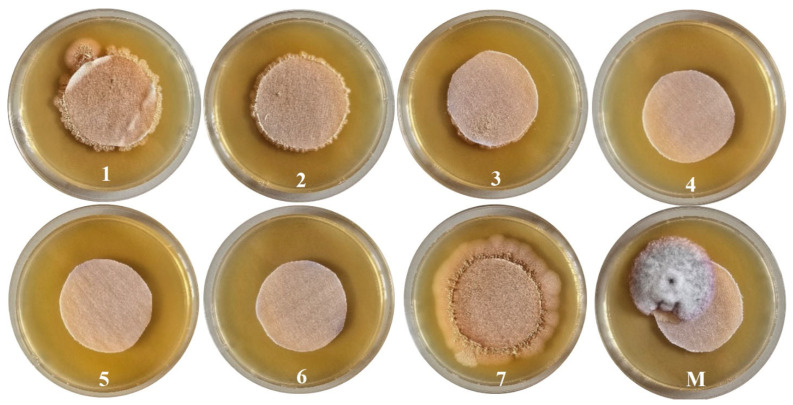
Samples after 96 h of incubation.

Antifungal testing first involved obtaining a fresh culture, and then the actual testing, which involved incubating the test samples for 4 h at 37 °C. After the incubation period, the sample plates were analyzed (Figure 12), and a score from 0 to 4 was given (Table 6), depending on the degree of growth present, as follows:

0: sample shows no fungal development;
1: traces of growth on the sample (less than 10%);
2: slight growth on the sample (between 10 and 30%);
3: medium growth on the sample (between 30 and 60%);
4: strong growth on the sample (between 60 and 100%).

The degree of fungal growth obtained when testing the antifungal activity of textile samples against Candida albicans is presented in Table 6.

We tested 8 textile materials: 7 samples functionalized with bioactive emulsions and 1 control (untreated cotton). The control (without pretreatment with CaCl_2_ and without active compounds) had significant fungal growth (score 3), confirming that the antifungal effect is directly associated with the applied treatment. All treated samples showed significantly higher antifungal activity than the control, confirming the effectiveness of the combination of natural compounds and pretreatment with CaCl_2_. Samples 4, 5, and 6 achieved complete inhibition (score 0), indicating that the formula and method of fixation on the textile support can be optimized for a high degree of efficiency. The differences between samples with a score of 1 and those with a score of 0 may come from: different proportions of essential oil and hydroalcoholic extract of thyme; different HMP content, which influences the fixation and release of active substances; and possible variations in the homogeneity of the application. However, grade 1 highlights microbial contamination on a surface area of less than 10%, so we can speak of high antifungal efficiency even in this case.

Samples 4, 5, and 6 (without fungal growth) had, according to the treatment, high pectin concentrations and lower proportions of hydroalcoholic extract compared to the other samples, which may indicate a denser matrix that retains the antifungal compounds. Samples 1, 2, 3, and 7, with a score of 1, may have had a lower dispersion or faster migration of the compounds, leaving exposed areas. At the same time, we can speak of a high degree of migration of the active compounds into the medium, inhibiting the growth of the strain on the culture medium. From this point of view, we can say that sample 7 had the best fixation of the treatment; it did not diffuse into the medium (nutrient medium with high humidity), based on the microbial growth in its vicinity. The ASTM G21 method scores the growth on the sample, not the surrounding “halo”; therefore, a weak/non-existent halo with a clean sample indicates predominantly contact activity, with little diffusion of the compounds into the medium, so a minimum degree of diffusion can be correlated with good fixation.

#### 3.2.7. Variations in Color of Functional Textiles

The use of hydroalcoholic extracts from thyme leaves in obtaining emulsions 1, 2, 3, 4, 6, and 7 led to a change in the color of the cellulose supports treated with these emulsions. The color parameters are presented in Table 7.

According to the results obtained and presented in Table 7, the color difference between the samples treated with emulsions and the untreated sample varies between 8.955 and 18.540, which means that the treated samples have completely different colors, not just shades of the same white. Thus, the smallest color difference compared to the untreated control sample is the cotton sample treated with the emulsion that does not contain the hydroalcoholic extract of thyme (8.955). The sample containing the largest amount of hydroalcoholic extract of thyme has the highest color intensity (1.74). The values for the color intensity decrease with the decrease in the amount of hydroalcoholic extract of thyme in the emulsion applied to the cotton textile supports. In addition, the brightness of the samples decreased with the application of the hydroalcoholic extracts of thyme on the cellulose supports. The chromatic parameters a* and b* were also modified following the application of the hydroalcoholic extract of thyme. With few exceptions, most samples show positive values for a and b, which means that the dominant color varies in the orange/warm red area.

#### 3.2.8. Analysis of Comfort Indices

The analysis of comfort indices is important in the case of obtaining functionalized textile supports, with potential topical applications.

**Figure 13:**
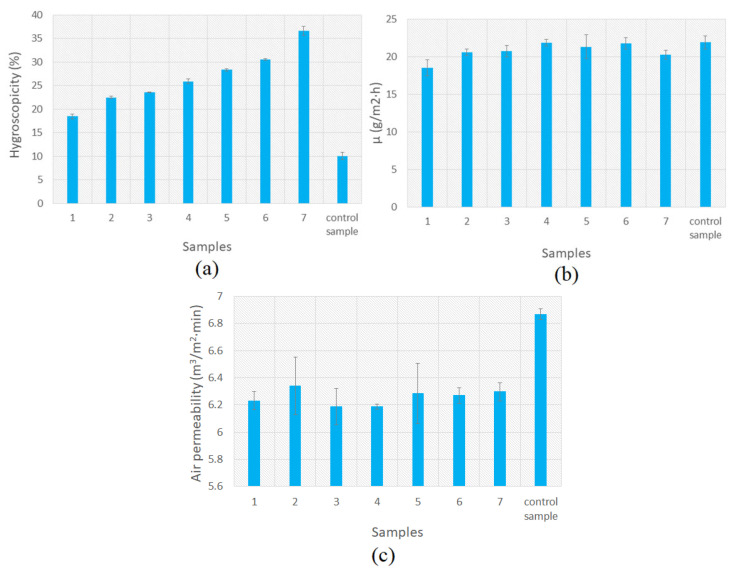
Comfort indices: (**a**) Hygroscopicity; (**b**) Water vapor permeability; (**c**) Air permeability. Each value in the figure represents the mean of three experiments ± the standard deviation.

According to the results obtained and presented in Figure 13a, the hygroscopicity decreases with increasing essential oil content and increases with increasing glycerin content, due to the ability to absorb atmospheric moisture. The essential oil constituting the discontinuous phase of the emulsion has a minor influence on the interaction with the surface and does not significantly influence the hygroscopicity of samples treated with emulsions. On the other hand, the pectin content also influences the hygroscopicity. With increasing HMP content, the hygroscopicity of samples treated with emulsions also increases. Pectin, due to its hydrophilic groups (hydroxyl and carboxyl), attracts water and thus contributes to the increase in the hygroscopicity of the obtained support, results also confirmed by the specialized literature [71,72,73]. Thus, the hydrophilic nature of pectin, as well as the presence of the hygroscopic plasticizer (glycerol) that favors water absorption and increases the moisture content of the obtained film explain the increase in hygroscopicity [74].

The water vapor permeability (Figure 13b) of the textile supports treated with emulsions decreases slightly compared to that of the untreated control sample. A possible explanation is the formation of a film on the surface of the textile support that limits the passage of water vapor, partially blocks the porosity of the fibers, and thus acts as a semipermeable barrier. Sample 1 shows the lowest water vapor permeability (the percentage difference compared to the standard sample is 15.5%, compared to the percentage difference in the other samples, which is <10%). The explanation may be given by its viscosity. Due to its viscous semi-solid consistency, emulsion 1 forms a more compact layer over the cellulose support and thus reduces the porosity of the fibers. These results are consistent with the mechanisms described in the literature on vapor transport through polymer films, according to which dense and non-porous films with a more compact physical structure limit the permeability of water vapor [75].

The air permeability of cellulose textile supports treated with active emulsions is not significantly affected (Figure 13c).

In conclusion, the breathability of the treated textile supports is not affected by the application of active emulsions.

## 4. Conclusions

This study investigated the potential of O/W bioactive pectin-based emulsions with oregano essential oil and hydroalcoholic extract of thyme to be used as a bioactive film when applied on cellulosic supports. Obtained bioactive textile supports were analyzed in terms of SEM-EDS elemental mapping, ATR FT-IR spectroscopy, biocompatibility assessment, antibacterial and antifungal activity assays, variations in color, and analysis of comfort indices. It was found that the textile supports treated with O/W emulsions are non-irritating, have antibacterial and antifungal properties, and can be used safely in topical applications. In conclusion, our results emphasize that the biocompatible, antibacterial, and antifungal textiles enriched with O/W emulsion are useful for skincare applications.

## Figures and Tables

**Table 1:** Working recipes.

Composition	Emulsions						
	1	2	3	4	5	6	7
Origanum essential oil (mL)	0.6	0.75	0.9	0.9	0.9	0.9	0.9
Pectin * (mL)	15	15	15	22.5	30	22.5	22.5
Polysorbate 80 (mL)	0.6	0.6	0.6	0.6	0.6	0.6	0.6
Vegetable glycerin (mL)	0.3	0.3	0.3	0.3	0.3	0.45	0.6
50:50 hydroalcoholic solution of thyme leaves (mL)	13.5	13.35	13.2	5.7	-	5.55	5.4

* 4% (*v*/*v*) citric pectin solution.

**Table 2:** Emulsion creaming index.

Emulsion	Days							
	0	2	4	6	8	10	12	14
1	100	100	100	100	100	100	100	100
2	100	100	100	100	100	100	100	100
3	100	100	100	100	100	100	100	100
4	100	100	100	100	100	100	100	100
5	100	100	100	100	100	100	100	100
6	100	100	100	100	100	100	100	100
7	100	100	100	100	100	100	100	100

**Table 3:** Film coverage (%).

Sample	1	2	3	4	5	6	7
Film coverage (%)	46.075 ± 15.9	49.699 ± 15.5	53.9215 ± 15.6	52.51 ± 14.3	58.311± 13.7	82.039 ± 6.4	90.845 ± 4.5

**Table 4:** Coating mass per unit area.

Sample	1	2	3	4	5	6	7
Coating mass (g/m^2^)	16.449 ± 0.8	14.601 ± 1.0	16.592 ± 0.6	17.160 ± 0.5	18.056 ± 1.0	25.398 ± 1.2	32.285 ± 1.4

**Table 5:** Radial inhibition zone (average ± standard deviation).

Samples	*S. aureus*	*E. coli*
1	4.66 ± 0.58 mm	2.66 ± 0.58 mm
2	2.66 ± 0.58 mm	2.00 ± 0 mm
3	3.66 ± 0.58 mm	2.33 ± 0.58 mm
4	2.33 ± 0.58 mm	1.66 ± 0.58 mm
5	3.66 ± 0.58 mm	3.66 ± 0.58 mm
6	3.33 ± 0.58 mm	0 mm
7	2.33 ± 0.58 mm	0 mm
Control sample (M)	0 mm	0 mm

**Table 6:** Degree of fungal growth.

Samples	*Candida albicans*	Score
1	<10%	1
2	<10%	1
3	<10%	1
4	0 (without growth)	0
5	0 (without growth)	0
6	0 (without growth)	0
7	<10%	1
Control sample (M)	30–60%	3

**Table 7:** Color parameters of treated samples.

Samples	K/S	L*	a*	b*	ΔE
Control sample	-	94.27	2.82	−8.47	-
1	1.74	88.68	−0.06	8.67	18.259
2	1.62	88.51	0.38	8.99	18.540
3	1.61	88.23	1.95	8.64	18.167
4	1.49	89.50	0.27	5.74	15.203
5	0.76	91.38	1.37	−0.12	8.955
6	1.31	87.39	2.56	5.86	15.900
7	1.25	89.13	1.72	4.73	14.210

## Data Availability

The data presented in this study are available on request from the corresponding author.
